# Epithelial-to-Mesenchymal Transition in Fibrosis: Concepts and Targeting Strategies

**DOI:** 10.3389/fphar.2021.737570

**Published:** 2021-09-07

**Authors:** Sara Lovisa

**Affiliations:** ^1^ Department of Biomedical Sciences, Humanitas University, Pieve Emanuele (MI), Italy; ^2^ IRCCS Humanitas Research Hospital, Rozzano (MI), Italy

**Keywords:** EMT, EMP, partial EMT, plasticity, fibrosis, epithelial-to-mesenchymal transition

## Abstract

The epithelial-to-mesenchymal transition (EMT), an embryonic program relaunched during wound healing and in pathological conditions such as fibrosis and cancer, continues to gain the attention of the research community, as testified by the exponential trend of publications since its discovery in the seventies. From the first description as a mesenchymal transformation, the concept of EMT has been substantially refined as an in-depth comprehension of its functional role has recently emerged thanks to the implementation of novel mouse models as well as the use of sophisticated mathematical modeling and bioinformatic analysis. Nevertheless, attempts to targeting EMT in fibrotic diseases are at their infancy and continue to pose several challenges. The aim of this mini review is to recapitulate the most recent concepts in the EMT field and to summarize the different strategies which have been exploited to target EMT in fibrotic disorders.

## EMT in 2021: Novel Refinements of an Old Concept

Since its first description in the embryogenesis work by Elizabeth Hay in the 1970s (Hay, 1968; Hay, 1995), the concept of epithelial-to-mesenchymal transition (EMT) has expanded from the field of development and has been investigated in the fields of wound healing, fibrosis, and cancer (Nieto et al., 2016). With an average of 5,000 primary papers published per year in the 2016–2019 period (Yang et al., 2020; Hamidi et al., 2021), the topic of EMT continues to gain the interest of the scientific community and to provide novel insights into this phenomenon both in physiology and in disease.

EMT is traditionally defined as a cellular and molecular process through which cells lose their epithelial identity, defined by apical–basal polarity and stable intercellular junctions, and acquire a mesenchymal phenotype including cytoskeletal and morphological rearrangements, acquisition of fibroblast-like gene expression profile, migratory capacity, and ability to produce the extracellular matrix (ECM) (Kalluri, 2009; Kalluri and Weinberg, 2009; Zeisberg and Neilson, 2009). However, recent studies as well as the fervent town hall discussions during the 2017 and 2019 meetings of The EMT International Association (TEMTIA) have clearly highlighted the need to revise and expand the traditional definition of EMT in order to embrace newly discovered features such as the partial activation of the program and the existence of a continuous spectrum of hybrid EMT phenotype rather than a binary E–M model and have therefore introduced and encouraged the use of the term “epithelial-to-mesenchymal plasticity” (EMP) (Yang et al., 2020).

The appreciation that EMT exists as a hybrid phenotype in a continuum of epithelial and mesenchymal traits has emerged from the construction of mathematical algorithms modeling the existence of multiple intermediate steps with various degrees of E or M states (Lu et al., 2013; Jolly et al., 2016; Tripathi et al., 2021), as well as from the pseudotemporal reconstruction of the EMT trajectory by single cell transcriptomics (Carstens et al., 2021; Deshmukh et al., 2021). The acquisition of this knowledge represents a great example of how crosstalk between different fields, such as mathematics and bioinformatics, can help in providing further understanding of the biology of EMT. In addition to the pure definition and the various criteria utilized to define this process, the functional role of EMT as defined by the type II and III classifications (Kalluri and Weinberg, 2009; Zeisberg and Neilson, 2009) requires to be updated in light of the most recent findings. In fact, the fibroblast-generating capacity of type II EMT during fibrosis has been rebutted (Ovadya and Krizhanovsky, 2015; Huang and Susztak, 2016; Lovisa et al., 2016), and the dispensability of type III EMT for metastasis has been questioned (Maheswaran and Haber, 2015; Brabletz et al., 2018).

This review aims to present the overview of recent concepts in EMT as well as novel insights as emerged from single cell transcriptomics, and to provide a summary of the strategies attempted to target EMT in the context of fibrotic diseases. So far, multiple approaches have been proposed to target EMT: from targeting the upstream inducing signaling pathways [which has been extensively reviewed in other recent reviews (Di Gregorio et al., 2020; Jonckheere et al., 2021)] to targeting EMT-transcription factors (TFs), promoting MET, and targeting EMT-induced vulnerabilities, the last being the strategy potentially leading to the most promising outcomes.

## EMT Classification: Type II and Type III Revisited

In these past years, the two major EMT paradigms permeating the fibrosis and cancer fields, which are, respectively, the capacity to generate fibroblasts (type II EMT) and the indispensability in the metastatic cascade (type III EMT), have been interrogated and partially revised. Historically, the outstanding question in the fibrosis field has been the origin of the myofibroblasts responsible for the scarring of the tissue. Candidate cellular origins include the activation of tissue-resident fibroblasts, the differentiation from bone marrow precursors, and the *trans*-differentiation of epithelial, mesothelial, and endothelial cells, macrophages, pericytes, and adipocytes into myofibroblasts (Plikus et al., 2021).

In light of this major question, EMT and the cognate process of endothelial-to-mesenchymal transition (EndMT) were initially identified as the mechanisms generating these fibrosis-associated myofibroblasts (Okada et al., 1997; Kim et al., 2006; Zeisberg M. et al., 2007; Zeisberg E. M. et al., 2007; Zeisberg et al., 2008; Flier et al., 2010). However, novel genetically engineered knock-out mouse models coupled with lineage tracing strategies clearly demonstrated that, at least in the context of kidney fibrosis, EMT does not directly generate myofibroblasts nor confers migratory capacity and that EMT cells still reside within the epithelial basement membrane in a partial EMT (pEMT) state (LeBleu et al., 2013; Grande et al., 2015; Lovisa et al., 2015). This pEMT represents a damage response of the injured renal epithelium, which substantially impairs epithelial functionality and regenerative capacity. In fact, pEMT triggers an arrest of the tubular epithelial cell cycle at the G2/M phase, therefore impeding the regenerative potential, and induces loss of the expression and functionality of membrane transporters critical for the absorptive capacity of the kidney (Lovisa et al., 2015). Moreover, the activation of the mesenchymal program leads to the acquisition of a pro-inflammatory secretome profile which in turn fuels immune infiltration and further promotes fibrosis (Grande et al., 2015; Lovisa et al., 2015). Similarly, the contribution of EndMT to the myofibroblast pool was determined as minor while having a significant impact on vascular integrity (LeBleu et al., 2013; Lovisa et al., 2020).

Mesothelial cells, which line pleural, peritoneal, and pericardial cavities, represent an example of physiologic pEMT as, in the basal condition, they phenotypically display epithelial features although concomitantly expressing mesenchymal markers such as vimentin, a remnant of their mesoderm-derived embryonic origin (Mutsaers et al., 2015). In the pathological condition, these cells undergo an EMT analogous process termed “mesothelial-to-mesenchymal transition” (MMT), which was found to be responsible for causing peritoneal fibrosis (Yáñez-Mó et al., 2003; Yang et al., 2003; Del Peso et al., 2008). The functional consequences of MMT as well as other types of mesenchymal *trans*-differentiation such as the one undergone by macrophages [termed “macrophage-to-myofibroblast transition” (Meng et al., 2016; Wang et al., 2017; Tang et al., 2020)] appear to still be unquestionably linked to the full transition and generation of fibrosis-associated myofibroblasts, with a consequent more direct impact on the generation of fibrosis (Koopmans and Rinkevich, 2018).

**FIGURE 1:**
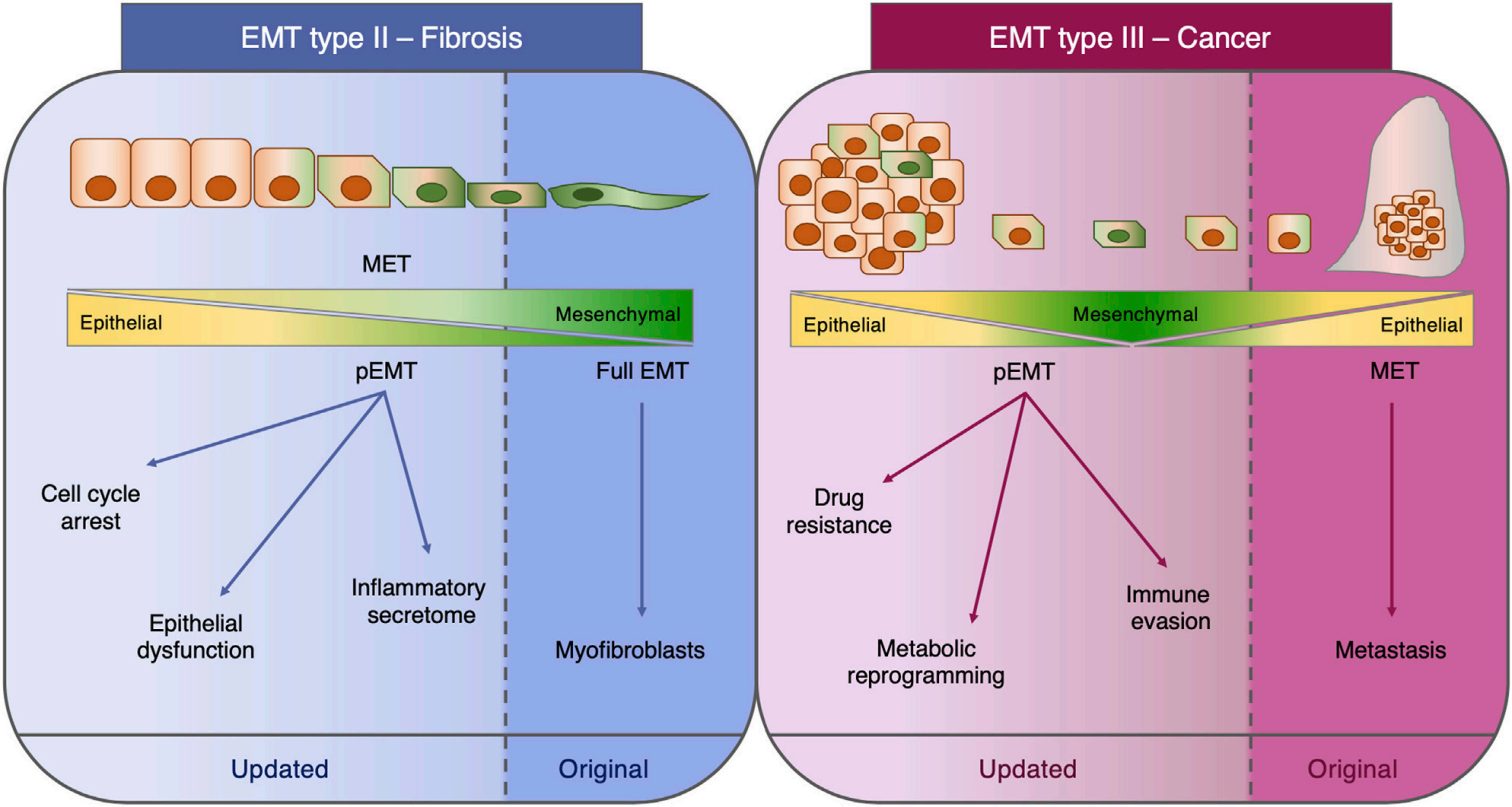
Type II and type III EMT. Schematic representation of the functional role of EMT in fibrosis (type II EMT) and cancer (type III EMT). For each class of EMT, both the original concept and the most recent findings are depicted. EMT: epithelial-to-mesenchymal transition; MET: mesenchymal-to-epithelial transition; pEMT: partial EMT.

The functional role of EMT in cancer has been similarly questioned. In fact, the metastasis dogma by which the metastasizing cells are those efficiently activating EMT to intravasate and extravasate and subsequently reverting to the epithelial state at distant sites through the process of mesenchymal-to-epithelial transition (MET) has been disproved at least in the context of breast (Fischer et al., 2015; Lourenco et al., 2020) and pancreatic cancers (Zheng et al., 2015; Chen et al., 2018; Carstens et al., 2021) and, since then, has been the subject of intense debate (Brabletz et al., 2018; Williams et al., 2019). The activation of EMT in cancer cells not only is connected with the metastatic potential but in these past years also has been clearly demonstrated to confer diverse advantageous properties such as resistance to chemotherapy, immune evading capacity, and rewiring of the cell metabolism (Kang et al., 2019; Lu and Kang, 2019; Bakir et al., 2020; Jia et al., 2021). All these concepts represent novel advancements in our knowledge on EMT which must be included in an updated classification (Figure 1). Moreover, these aspects highlight how dynamic and complex the EMT is and how the generation of new *in vivo* models coupled with technology advancement can provide a deeper understanding of this phenomenon which sometimes may lead to unexpected findings with respect to the original concepts.

## Understanding EMT by Single Cell Transcriptomics

Our comprehension of the dynamics of EMT has significantly advanced thanks to the introduction of technologies such as single cell RNA-sequencing (scRNA-seq). A first study employing scRNA-seq and pseudospatial trajectory reconstruction of epithelial cells undergoing spontaneous or TGFβ-induced EMT revealed that the EMT is a transcriptional continuum of epithelial–mesenchymal states (McFaline-Figueroa et al., 2019). Interfering with signaling pathways by inhibiting transcription factors (TFs) or receptors impeded the progression along the EMT and caused cells to accumulate at defined points in the EMT continuum, thus revealing the existence of regulatory checkpoints (McFaline-Figueroa et al., 2019). This observation indicated that disabling key signaling pathways could enrich a particular gene expression profile, therefore giving the impression of a stable E/M intermediate phenotype.

By coupling scRNA-seq and mathematical modeling to a time course experiment of TGFβ-induced EMT in the MCF10A breast cell line, a recent study mapped the molecular changes and signaling cascades occurring during EMT progression (Deshmukh et al., 2021). Fundamental findings are the fact that many EMT regulatory pathways (Notch, Shh, Wnt, PI3K/Akt) were found to be activated simultaneously, possibly indicating that a crosstalk among multiple signaling pathways may occur in a temporal manner and that the rate of progression through EMT was not the same for all the cells, indicating a temporal heterogeneity in the activation of EMT. Even after 8 days of TGFβ treatment, half of the analyzed cells were in the hybrid E/M state, and the pseudotime analysis revealed the presence of twenty distinct EMT clusters (Deshmukh et al., 2021).

One recurrent issue in analyzing EMT at the transcriptional level has been the impossibility to distinguish the mesenchymal signature of the epithelium from the one in the stromal compartment, due to the promiscuous expression of the markers analyzed. Recently, a computational framework to decouple the true EMT signature of epithelial cells from the stromal mesenchymal signature in bulk RNA-seq data has been developed to characterize EMT across different types of tumors (Tyler and Tirosh, 2021). This method revealed that the expression of the classical EMT-transcription factors (except *SNAI2*) is very high in cancer-associated fibroblasts and therefore should not be used as a marker of partial EMT in bulk analysis. Certainly, a similar bioinformatic approach would be desirable for the bulk RNA-seq dataset of fibrotic disorders to analyze pEMT without the confounder of the fibrotic stroma.

A common concept emerging from different studies employing scRNA-seq is represented by the fact that the pEMT profile is highly context-specific (Cook and Vanderhyden, 2020; Tyler and Tirosh, 2021). This concept was, for example, highlighted by a multiplexed scRNA-seq of EMT time course induction in four different cell lines, using three distinct inducers (TGFβ1, TNF, EGF) and also including the analysis of EMT reversion by removal of the inducing signal (Cook and Vanderhyden, 2020). Pseudotemporal trajectories confirmed that EMT is not just a linear progression but rather a multistep process characterized by a series of discrete transcriptional events. Surprisingly, the activity of TFs was also remarkably context specific, and TFs that have been implicated in EMT but are not the traditional core EMT-TFs were found differentially regulated in a context-specific manner (Cook and Vanderhyden, 2020). A combined bioinformatic and mathematical analysis on the same time-series scRNA-seq allowed to construct a context-specific EMT gene regulatory circuit (GRC) from transcriptomics data to identify activity dynamics of EMT-TFs (Ramirez et al., 2020). Although most of these scRNA-seq studies were conducted using tumor cell lines and, therefore, it cannot be assumed that the same findings apply to non-transformed epithelial cells activating pEMT as part of their injury-induced damage response, these studies provided novel insights into the dynamics of EMT which are worth to be taken into consideration when studying EMT in the context of fibrosis.

## Direct Targeting of EMT-TFS: Genetic Deletion and Small Molecule Inhibitors

The most compelling evidence that inhibition of EMT-driving TFs is an effective strategy for reducing fibrosis has been generated by using genetically engineered knock-out mouse models. In fact, renal epithelial cells’ conditional deletion of the *Twist1* or *Snail1* genes, encoding, respectively, Twist1 and Snail EMT-TFs, using two distinct epithelial-driven Cre/lox models (γGT-Cre and Cdh16-Cre), proved to be effective in inhibiting the process of EMT and led to substantial reduction of kidney fibrosis (Grande et al., 2015; Lovisa et al., 2015). Epithelial-specific inducible activation of Snail was necessary and sufficient to induce fibrosis which could be reversed by deactivating or silencing Snail (Grande et al., 2015). Genetic deletion of these EMT-TFs efficiently reduced ECM deposition, myofibroblast accumulation, and immune infiltration and led to a significantly improved tubular epithelial functionality and regenerative capacity, therefore demonstrating that EMT inhibition leads to both epithelial recovery and suspension of the paracrine effect on mesenchymal and immune cells (Grande et al., 2015; Lovisa et al., 2015). A similar paracrine effect exerted on fibroblasts by epithelial cells undergoing EMT has been reported in lung fibrosis (Hill et al., 2019; Yao et al., 2019). Mice with conditional deletion of Snail in hepatocytes using the albumin-Cre model also display reduced ECM and immune infiltration during hepatic fibrosis, with no direct effects on hepatic stellate cell activation (Rowe et al., 2011).

Similarly, conditional deletion of Twist1 or Snail in endothelial cells (using Cdh5- and Tie1-Cre models) was recently shown to inhibit the cognate process of EndMT and protect from kidney fibrosis by limiting vascular leakage and the downstream hypoxia-driven metabolic rearrangements (Balzer and Susztak, 2020; Lovisa et al., 2020). Tie2-driven conditional deletion of Twist1 in the endothelium was also shown to be associated with reduction of lung fibrosis (Mammoto et al., 2013; Mammoto et al., 2016; Mammoto et al., 2018), while Tie2-driven deletion of Snail led to embryonic lethal vascular defects (Wu et al., 2014).

Based on this evidence, a pharmacological approach directly targeting EMT-TFs would theoretically represent an efficient strategy to inhibit EMT. Although, being transcription factors, Twist and Snail are usually considered undruggable and their pharmacological targeting remains challenging, there are reports of compounds derived from natural products that can target Twist, Snail, and Zeb1 (Pei et al., 2017; Avila-Carrasco et al., 2019; Feng et al., 2020). It is to be noted, however, that most of these studies attempt to target cancer-related EMT and these inhibitors are not fully specific for these TFs.

Alternative strategies include targeting protein effectors responsible for the post-translational control of TF stability. One example is a recent study reporting a small molecule which, by disrupting Snail–CBP/p300 interaction, promotes Snail proteasomal degradation and therefore reverses Snail-induced EMT and its associated tumor invasion and metastasis (Li et al., 2020). In the context of fibrosis, a recent study identified triptolide, a small molecule inhibitor targeting MEX3C, the E3 ligase responsible for PTEN polyubiquitination, as an EMT inhibitor (Li et al., 2019). High glucose–induced polyubiquitination of PTEN triggers phosphatase activity and favors the dephosphorylation of Twist and Snail, which in turn stabilizes these two TFs and induces EMT. The authors showed that triptolide treatment *in vitro* was able to reduce the glucose-induced protein expression of both Twist and Snail and successfully inhibited EMT and kidney fibrosis in both spontaneous and experimentally induced *in vivo* models (Li et al., 2019).

Recent advances in the use of nanoparticles and microvesicles such as exosomes have proven the efficacy and therapeutic application of siRNA delivery, and therefore, this could potentially be exploited as a strategy to target EMT, although the lack of cell specificity could represent a serious concern with this type of approach. In fact, aspects to be taken into consideration are the mutual interdependency of the EMT-TFs and their EMT-independent functions (Stemmler et al., 2019). Although in general these TFs are not expressed in adult tissues, there is evidence supporting their necessity in the adult process like Slug required in the process of cutaneous wound re-epithelialization (Hudson et al., 2009) and Twist2 expressed by a multipotent cell population generating cardiomyocytes in the adult heart (Min et al., 2018). Therefore, the extent to which these EMT-TFs are required for adult tissue homeostasis, cell identity, and fate determination is not completely known, and this might pose an obstacle for anti-EMT therapeutic strategies not targeted to a specific cell type.

## Reversing EMT: The Mesenchymal-to-Epithelial Transition

The mesenchymal-to-epithelial transition (MET) is a process employed during embryonic development to generate epithelia (Pei et al., 2019). Induction of MET has been associated with amelioration of fibrosis. In fact, reversion of TGFβ-induced EMT in tubular epithelial cells (Zeisberg et al., 2003) and induction of MET in fibroblasts in the injured kidney (Zeisberg et al., 2005) were shown to result in reduction of fibrosis and promotion of kidney regeneration. Induction of MET by treatment with BMP-7 was shown to improve *in vivo* fibrosis in renal, cardiac, and intestinal models (Zeisberg et al., 2003; Zeisberg et al., 2007; Flier et al., 2010), and treatment with a BMP agonist was indeed able to revert established fibrosis (Sugimoto et al., 2012). These observations are completely in line with the requirement of MET for the reprogramming of fibroblasts into pluripotent stem cells (Li et al., 2010) and the role of BMPs in driving the initiation of such MET-mediated reprogramming (Samavarchi-Tehrani et al., 2010).

Induction of MET in cardiac fibroblasts by stimulation of the p53 pathway induced the regeneration of functional vessels, through a process called “mesenchymal-to-endothelial transition” (MEndT) (Miyake and Kalluri, 2014; Ubil et al., 2014). MEndT contributes to neovascularization in the injured heart, and its induction improved cardiac function (Ubil et al., 2014; Dong et al., 2020). Treatment with the small molecule RITA, which inhibits ubiquitin-mediated p53 degradation and enhances p53 signaling, increased MEndT, reduced cardiac fibrosis, and improved cardiac function, therefore mechanistically proving that p53-mediated activation of MEndT in cardiac fibroblasts is able to limit cardiac injury (Ubil et al., 2014).

Novel insights into mechanisms of MET are inferred from single cell transcriptomics (Cook and Vanderhyden, 2020). EMT and MET were investigated by scRNA-seq in four different cell lines by induction with TGFβ, TNF, or EGF for 7 days, followed by 3 days of withdrawal time which was sufficient to almost completely revert cells transcriptionally to the epithelial state. Analysis of the time-dependent shifts in the gene expression profile showed that while it is true that stimulus withdrawal led to MET reversibility, it is also clear that the trajectory of changes in the reversion expression profile did not match that of the EMT induction (Cook and Vanderhyden, 2020). Further bioinformatics and mathematical modeling confirmed that EMT and MET trajectories have two distinct paths which do not overlap (Ramirez et al., 2020). This certainly indicates that EMT and MET are not perfectly symmetric processes and MET should not be oversimplified as the equal and opposite process of EMT.

It was reported that alternates of EMT–MET are necessary to induce pluripotency in somatic cell reprogramming, so that EMT is necessary to favor the subsequent MET (Liu et al., 2013; Li et al., 2017). This implies two considerations: 1) targeting EMT might then not be strategic in the attempt to favor MET-mediated regeneration over injury and fibrosis and 2) if some degree of EMT favors the subsequent MET in pluripotent reprogramming, one could argue that this predisposition could potentially occur also in the context of injury. A recent study on mechanisms of cardiac repair shows that dedifferentiation and activation of an EMT-like program in adult cardiomyocytes, induced by the ectopic reactivation of ERBB2 and mediated by YAP, are indeed necessary for migration and subsequent redifferentiation of cardiomyocytes at the injured site (Aharonov et al., 2020; González-Iglesias and Nieto, 2020). A similar requirement for YAP-induced EMT in hepatocytes was reported promoting liver regeneration (Oh et al., 2018). Therefore, why injury-induced EMT fails to successfully prime cells for reprogramming and regeneration in some contexts, such as the kidney, is clearly an open question that requires deeper investigation.

## Targeting EMT-Dependent Metabolic Vulnerabilities

The comprehension of the functional role of EMT beyond the mere generation of fibroblasts is potentially opening the opportunity to target EMT from a different perspective consisting in the identification of the EMT-induced cellular vulnerabilities mediated by druggable targets.

Disruption of the tissue metabolic homeostasis represents a hallmark of fibrosis, and targeting this metabolic dysregulation has started to emerge as a potential strategy for fibrosis treatment (Zhao et al., 2020). Defective fatty acid oxidation (FAO) was shown to induce renal fibrosis, and FAO inhibition provokes features of dedifferentiation, namely, the expression of mesenchymal markers, in the renal epithelium (Kang et al., 2015). FAO decrease and the consequent lipid accumulation were shown to induce an EMT expression profile in renal epithelial cells *in vitro* (Xu et al., 2014). FAO improvement by overexpression of the transcription factor PPARGC1A, which regulates the expression of all FAO rate-limiting enzymes, or by pharmacological treatment with fenofibrate was capable of protecting renal epithelial cells from TGFβ-induced dedifferentiation toward a mesenchymal profile (Kang et al., 2015). Conversely, *in vivo* inhibition of EMT during fibrosis was able to restore FAO and metabolic homeostasis, in association with improved epithelial health and functionality (Lovisa et al., 2015).

Reversion of the TGFβ-induced PPARγ inhibition by curcumin treatment was shown to inhibit EMT and ameliorate TNBS-induced intestinal fibrosis (Xu et al., 2017). Treatment with a PPARγ antagonist reverted the EMT inhibitory effect of curcumin, therefore further highlighting the existence of the FAO-EMT axis and the anti-fibrotic effects of the PPARγ agonists.

The interdependency of FAO metabolism and mesenchymal transition was also highlighted in the context of EndMT. Suppression of FAO by endothelial conditional deletion of the FAO enzyme CPT2 spontaneously induced amplification of the embryonic EndMT which resulted in the thickening of the cardiac valves and provoked EndMT with consequent abnormal vascular permeability in the kidney, spleen, and lung (Lovisa and Kalluri, 2018; Xiong et al., 2018). Inhibition of EndMT was proven to ameliorate fibrosis and restore the metabolic functionality of the kidney (Lovisa et al., 2020). In fact, the vascular leakage caused by the process of EndMT leads to a cascade of events characterized by a hypoxia-induced epithelial upregulation of the c-Myc transcription factor, which in turn is responsible for a glycolytic switch of the renal metabolism which normally heavily depends on FAO (Lovisa et al., 2020). The increase in glycolysis was proved to be detrimental as the treatment with the glycolysis inhibitor 3-bromopyruvate ameliorated tissue fibrosis (Lovisa et al., 2020; Yu et al., 2021). Moreover, genetic or pharmacological targeting of c-Myc by treatment with the JQ1 inhibitor reduced fibrosis, preserved the epithelial parenchyma, and restored the metabolic homeostasis (Lovisa et al., 2020). Whether inhibition of glycolysis is able to reduce EMT was not investigated; however, it is possible as EMT cells switch their metabolism from oxidative phosphorylation to glycolysis and scRNA-seq confirmed the downregulation of genes of the mitochondrial oxidative phosphorylation (Deshmukh et al., 2021).

Additionally, high glucose itself was shown to induce EMT in renal tubular epithelial cells (Li et al., 2020). Inhibition of sodium-glucose cotransporter 2 (SGLT2) suppressed glucose-induced EMT and decreased renal fibrogenesis (Li et al., 2020). SGLT2 suppression in tubular epithelial cells was also able to suppress EndMT of the peritubular capillaries (Li et al., 2020), further highlighting the existence of an epithelial–endothelial crosstalk during tissue injury (Balzer and Susztak, 2020).

To unravel the interdependency between metabolism and EMP, the first step would be to perform a comprehensive analysis of the metabolome of EMT during fibrosis, including the analysis of single cell metabolism along the continuum of the EMT spectrum. Considering that targeting EMT through metabolic inhibitors has gained great attention in the cancer field (Ramesh et al., 2020), it would be logical to argue that this approach might be translated as well in fibrotic diseases, with repurposed metabolic inhibitors potentially becoming a valuable strategy to target fibrosis.

## Concluding Remarks

Fibrosis is the final outcome of a cascade of events participating in an uncontrolled wound healing response which causes an exaggerated accumulation of ECM, eventually leading to tissue scarring and organ failure (Zeisberg and Kalluri, 2013; Distler et al., 2019; Henderson et al., 2020). Fibrosis can affect any organ, and it is estimated to be responsible for up to 45% of the deaths worldwide, therefore representing a major global healthcare burden which cannot be further ignored (Henderson et al., 2020). The gigantic effort to understand mechanisms of fibrosis pathogenesis using experimental models and cutting-edge techniques such as single cell sequencing has not yet been translated into effective clinical trials. The gap between the promising results obtained with *in vivo* experimental models and the failure faced when they are clinically translated is enormous and demands immediate action.

In the context of identifying cellular drivers of fibrosis, EMT was thought to be the major mechanism causing the accumulation of myofibroblasts (Kalluri and Weinberg, 2009). Although this might be true *in vitro*, where treatment with inflammatory cytokines forces epithelial cells to transition to an almost full mesenchymal phenotype, it appears that this is not the case *in vivo*. Instead, *in vivo* EMT cells reside in a hybrid partial EMT state which functionally participates in causing a detrimental damage response of the injured tissue. Although being in principle highly valuable (Table 1), targeting EMT has emerged as a challenging task. Multiple factors contribute to this difficulty in developing effective anti-EMT strategies, including the dynamic transition through the hybrid state, the theoretical infinity of the E/M intermediates, its orchestration mainly at the transcriptional level, and the co-existence of multiple and partially overlapping EMT-inducing pathways.

**TABLE 1:** Summary of the different strategies to target EMT in fibrosis.

Targeting strategy	Molecular targets		Approach	References
EMT-inducing pathways	TGFβ, Hedgehog, Hippo, Wnt, and Notch signaling pathways		Genetic deletion, antagonists, small molecule inhibitors, miRNAs, natural compounds	Avila-Carrasco et al. (2019); Di Gregorio et al. (2020); Jonckheere et al. (2021)
EMT-transcription factors	Twist, Snail		Genetic deletion in epithelial and endothelial cells	Rowe et al. (2011); Mammoto et al. (2013); Wu et al. (2014); Grande et al. (2015); Lovisa et al. (2015); Mammoto et al. (2016); Mammoto et al. (2018); Lovisa et al. (2020)
	Snail–CBP/p300 interaction		Small molecule inhibitor	Li et al. (2020) (cancer)
	MEX3C-mediated, PTEN-induced Twist and Snail phosphorylation		Small molecule inhibitor	Li et al. (2019)
	Twist, Snail, Slug, Zeb1		Natural compounds	Pei et al. (2017); Avila-Carrasco et al. (2019); Feng et al. (2020)
MET	BMP-7		Agonist	Zeisberg et al. (2003); Zeisberg et al. (2005); Zeisberg et al. (2007); Flier et al. (2010); Sugimoto et al. (2012)
	Ubiquitinated p53		Small molecule inhibitor	Ubil et al. (2014)
EMT-related metabolic vulnerabilities	FAO	PPARGC1A, PPARα, PPARɣ	Genetic induction, agonist, natural compound	Kang et al. (2015); Xu et al. (2017)
	Glycolysis	c-Myc	Genetic deletion in epithelial cells, transcriptional repression	Lovisa et al. (2020)
		HK2	Inhibitor	Lovisa et al. (2020); Yu et al. (2021)
		SGLT2	Inhibitor	Li et al. (2020)

EMT: epithelial-to-mesenchymal transition; MET: mesenchymal-to-epithelial transition; FAO: fatty acid oxidation.

EMT has been recently looked at as an attractive target in oncology (Marcucci et al., 2016). This interest is not quite reflected in the fibrosis field, but certainly cross-communication between these two areas of investigation could improve and optimize the effort toward targeting EMT. The interest of the cancer scientific community on EMT mainly regards targeting the possible metabolic alteration accompanying EMT (Ramesh et al., 2020). Focusing the attention on the EMT-induced vulnerabilities might indeed represent a promising strategy, which would circumvent all the difficulties associated with directly targeting the transcriptional drivers of EMT. Moreover, this strategy would open the possibility for therapeutic repurposing of metabolic drugs to fibrotic diseases. Persevering in our effort to better understand the biological basis of EMT will certainly help in identifying novel routes for therapeutic intervention.
